# Application of Integrated Emergency Care Model Based on Failure Modes and Effects Analysis in Patients With Ischemic Stroke

**DOI:** 10.3389/fsurg.2022.874577

**Published:** 2022-04-05

**Authors:** Yuying Yang, Qing Chang, Jing Chen, Xiangkun Zou, Qian Xue, Aixia Song

**Affiliations:** ^1^Stroke Center Office, The First Affiliated Hospital of Hebei North University, Zhangjiakou, China; ^2^Department of Neurology, The First Affiliated Hospital of Hebei North University, Zhangjiakou, China; ^3^Imaging Department, The First Affiliated Hospital of Hebei North University, Zhangjiakou, China; ^4^Information Section, The First Affiliated Hospital of Hebei North University, Zhangjiakou, China

**Keywords:** acute ischemic stroke, failure modes and effects analysis, integrated emergency care model, emergency procedures, clinical outcomes

## Abstract

**Purpose:**

To explore the application value of an integrated emergency care model based on failure modes and effects analysis (FMEA) in patients with acute ischemic stroke (AIS).

**Methods:**

According to the convenience sampling method, 100 patients with AIS who visited the emergency department in our hospital from October 2018 to March 2019 were randomly selected as the control group and received routine emergency care mode intervention. Another 100 AIS patients who visited the emergency department from April to October 2019 were selected as the intervention group and received the integrated emergency care model based on FMEA. The total time spent from admission to completion of each emergency procedure [total time spent from admission to emergency physician reception (T_0−1_), total time spent from admission to stroke team reception (T_0−2_), total time spent from admission to imaging report out (T_0−3_), total time spent from admission to laboratory report out (T_0−4_), and total time spent from admission to intravenous thrombolysis (T_0−5_)] was recorded for both groups. The clinical outcome indicators (vascular recanalization rate, symptomatic intracerebral hemorrhage incidence, mortality rate) were observed for both groups. The National Institutes of Health Stroke Scale (NIHSS) score and Barthel score were evaluated for both groups after the intervention. The treatment satisfaction rate of the patients was investigated for both groups.

**Results:**

The total time of T_0−1_, T_0−2_, T_0−3_, T_0−4_, T_0−5_ in the intervention group (0.55 ± 0.15, 1.23 ± 0.30, 21.24 ± 3.01, 33.30 ± 5.28, 44.19 ± 7.02) min was shorter than that of the control group (1.22 ± 0.28, 4.01 ± 1.06, 34.12 ± 4.44, 72.48 ± 8.27, 80.31 ± 9.22) min (*P* < 0.05). The vascular recanalization rate in the intervention group (23.00%) was higher than that in the control group (12.00%) (*P* < 0.05). There was no statistical significance in the symptomatic intracerebral hemorrhage incidence and mortality rate in the two groups (*P* > 0.05). After intervention, the NIHSS score of the intervention group (2.95 ± 0.91) was lower than that of the control group (6.10 ± 2.02), and the Barthel score (77.58 ± 7.33) was higher than that of the control group (53.34 ± 5.12) (*P* < 0.05). The treatment satisfaction rate in the intervention group (95.00%) was higher than that of the control group (86.00%) (*P* < 0.05).

**Conclusion:**

Through FMEA, the failure mode that affects the emergency time of AIS patients is effectively analyzed and the targeted optimization process is proposed, which are important to enhance the efficiency and success rate of resuscitation of medical and nursing staff and improve the prognosis and life ability of patients.

## Introduction

Acute ischemic stroke (AIS) is the most prevalent type of stroke in clinical practice, accounting for more than 80% of strokes, and it is a major public health problem that threatens national health in my country (1). The disease is mostly secondary to systemic diseases such as hypertension (2), atherosclerosis (3), heart disease (4) and coagulation dysfunction (5), and is characterized by a high recurrence rate, high disability rate, high complication rate and high mortality rate. The key to AIS treatment is to restore blood flow to the occluded vessel and save the ischemic penumbra within the treatment time window (i.e., 3 to 4.5 h after the onset of stroke) (6). Intravenous thrombolysis is the highest-level recommendation in current international guidelines for the treatment of ultra-early AIS (7). However, due to the low recognition of early AIS by the public in China, the ineffective pre-hospital treatment by medical and nursing staff, and the delay in in-hospital emergency care, only about 21.5% of patients arrive at the emergency department within 3 h of onset, and the average time from admission to thrombolytic drug treatment is 116 min, of which only about 9% of patients complete thrombolytic treatment within 1 h after admission, much lower than 50% in the United States. And it has been reported that after thrombolysis, 6 % of patients are still at risk of symptomatic intracranial hemorrhage, and 70% of patients still have symptoms of varying degrees of disability (8, 9). The treatment situation of AIS patients in my country is very severe, and the treatment efficiency is not optimistic. It is urgent to formulate an optimization plan to effectively shorten the delay in the hospital as soon as possible.

Failure modes and effects analysis (FMEA) is a commonly used method in foreign countries to actively assess risks to improve the quality of medical management. It integrates failure mode and effect analysis, hazard analysis and critical control points, and root cause analysis. By prospectively quantifying and evaluating the possible failure links of a first aid process, analyzing its failure causes and effects, and formulating targeted solutions accordingly, its essence is a continuous quality improvement process. It has been widely used in medication guidance, diagnosis and treatment process, risk management, surgery and nursing operations, etc., but it is rarely used in my country's emergency nursing model (10, 11). This study explores the application value of the integrated emergency care model based on FMEA in patients with AIS, in order to provide an important theoretical basis for optimizing the in-hospital emergency mode of AIS patients and shortening the delay time of in-hospital emergency care.

## Materials and Methods

### Research Object

According to the convenience sampling method, 100 patients with AIS who visited the emergency department in our hospital from October 2018 to March 2019 were randomly selected as the control group. Another 100 AIS patients who visited the emergency department from April to October 2019 were selected as the intervention group. Inclusion criteria: Those with a suspected AIS diagnosis were screened by the Los Angeles prehospital stroke screen (LAPSS) (12) before diagnosis; During the implementation of the emergency procedures, those who were diagnosed by imaging examinations and met the diagnostic criteria of the Chinese Stroke Society (13) for AIS; Age ≥18 years; time from onset to consultation ≤ 3–4.5 h; those who met the requirements of 3.0–4.5 h for intravenous thrombolysis for AIS patients set by the American Heart and Stroke Association (14); patients or their families who had signed relevant informed consent. Exclusion criteria: Those who had been transferred to our hospital after completion of examination or diagnosis in another hospital; those who had undergone major surgical operations in the past 2 weeks; those with other serious primary diseases or mental illnesses; those with absolute contraindications to thrombolysis therapy. Statistical software was used to analyze the general data of the two groups of patients, and there was no statistical difference, which was comparable (*P* > 0.05). As seen in Table 1.

**Table 1:** General information of two groups of patients.

**Information**	**Control group (*n* = 100)**	**Intervention group (*n* = 100)**	***t*/χ^2^**	***P***
Age (M ± SD, years old)	67.96 ± 6.95	66.58 ± 7.24	1.375	0.171
Male [*n* (%)]	49 (49.00)	54 (54.00)	0.501	0.479
History of hypertension [*n* (%)]	56 (56.00)	52 (52.00)	0.322	0.570
History of diabetes [*n* (%)]	33 (33.00)	35 (35.00)	0.089	0.765
History of hyperlipidemia [*n* (%)]	29 (29.00)	24 (24.00)	0.642	0.423
History of coronary heart disease [*n* (%)]	13 (13.00)	15 (15.00)	0.166	0.684
History of atrial fibrillation [*n* (%)]	17 (17.00)	18 (18.00)	0.035	0.852
History of transient cerebral ischemic attack [*n* (%)]	5 (5.00)	7 (7.00)	0.355	0.552
History of stroke [*n* (%)]	12 (12.00)	10 (10.00)	0.204	0.651
History of smoking [*n* (%)]	29 (29.00)	33 (33.00)	0.374	0.541
History of drinking [*n* (%)]	19 (19.00)	22 (22.00)	0.276	0.599

### Care Methods

Control group: intervention with routine emergency nursing mode was given. It mainly included emergency triage nurse pre-screening, notification of physician consultation, preliminary diagnosis and treatment of patients, various auxiliary examinations for patients, doctor's assessment of patient's condition, doctor-patient conversation to decide whether to implement intravenous thrombolysis, preparation for thrombolysis treatment, interfacing with neurology/neurosurgery and other routine treatment procedures.

**Figure 1:**
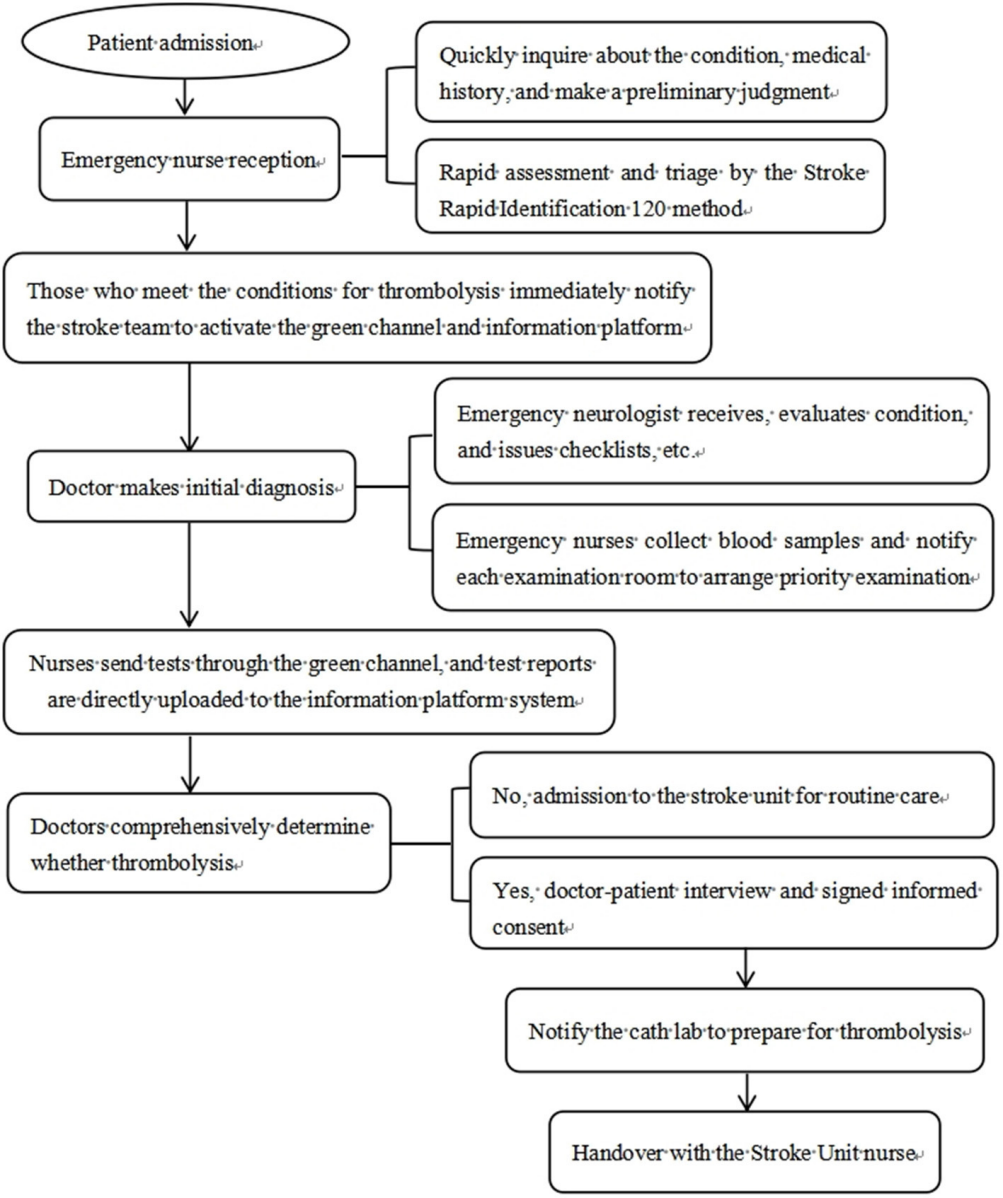
Flow chart of the integrated emergency care model based on FMEA of AIS patients.

Intervention group: intervention with integrated emergency care model based on FMEA was given. Step I: a 10 person stroke emergency care team was formed. Nursing experts with rich experience and strong nursing ability were included in emergency department, neurology department, radiology department, laboratory department and other departments. Step II: a total of 8 special seminars for a month was carried out. During the meeting, the stroke team was given training on FMEA management method, AIS emergency procedures and other related knowledge. During this period, the stroke team analyzed the causes of failure and the risk priority number (RPN) for possible failure modes in the AIS emergency procedure, and indicated the presence of high-risk links when the RPN score was >125. RPN was the product of event severity (S), occurrence (O) and detection indexes (D). After analysis, there were the following 6 high-risk links in the emergency process of AIS patients in our hospital: (1) Preliminary triage, (2) Families payment, examination appointment and medication collection, (3) Families send for examinations, (4) Doctor-patient communication to decide whether to thrombolize, (5) Emergency nurses dispense drugs, (6) Thrombolysis preparations. The FMEA of each high-risk link as shown in Table 2. Step III: For the above-mentioned high-risk links, priority should be given to developing targeted and optimized care processes to reduce the harm of failure modes. The corresponding optimization measures for each high-risk link as shown in Table 3. The problems that emerged during the implementation of the optimized process were discussed and improved to ensure continuous improvement of the quality of emergency care. The optimized integrated emergency care model of this study as shown in Figure 1.

**Table 2:** FMEA for emergency care of AIS patients.

**High-risk** **link**	**Failure modes**	**Failure reasons**	**Risk analysis**			
			**S**	**O**	**D**	**PRN**
1	Inappropriate assessment by triage nurses	The triage nurses were inexperienced, lacked the concept of “time window,” and had no specific clinical pathways and plans	7.20	8.40	7.50	453.60
2	Round-trip delay, information blocking	Departments were far apart, families were not familiar with the layout of the hospital and the diagnosis and treatment process, the hospital lacked signs and the information platform was weak, and the hospital stipulated that payment, examination appointment, and medicine need to queue up	8.20	7.80	7.50	479.70
3	Forwarding or pending delay	Lack of AIS green channels, inspections and reports did not specify that AIS would be prioritized	7.50	8.40	7.60	478.80
4	Delayed or poor communication	Untimely or inadequate health education and conservative attitude of physicians toward thrombolysis	7.80	9.00	7.80	547.56
5	Dispensing medicines not on time	There were many cases and diseases in the emergency department, and nurses lacked the concept of “time window”, and there was no special clinical path and plan	8.80	7.50	7.50	495.00
6	Delayed transfer to the cath lab and delayed thrombolysis preparation	Cath lab might be in use, delayed arrival of medical staff, delayed preparation for thrombolysis	8.20	7.60	9.00	560.88

**Table 3:** Corresponding optimization measures for each high-risk link of AIS patients.

**High-risk link**	**Optimization measures**
1	Regularly train and assess the knowledge of AIS and thrombolysis for emergency nurses, and formulate the “Emergency AIS Thrombolysis Process Emergency Plan”.
2	Set up functions such as “one-click payment, appointment inspection, and report viewing” on the hospital's WeChat platform, and taking medicines is carried out by nurses in the hospital.
3	Set up a green channel for AIS transfer, the patient's delivery is carried out by professional nurses trained in transfer, and the specimen delivery should be labeled as “stepped up”.
4	At the time of admission, the possibility of thrombolysis is informed and relevant knowledge is popularized. The electronic bulletin screen in the consultation room broadcasts relevant knowledge and notifications of thrombolysis to help patients or their families understand and decide as soon as possible.
5	Set up a special post for thrombolysis nurses to be responsible for thrombolysis treatment of patients.
6	A spare cath lab and a green channel for AIS thrombolysis are set up. After confirming the patient's thrombolysis, open the cath lab and the green channel immediately, and transfer the patient to the cath lab accompanied by doctors and nurses.

### Observation Index

The total time spent from admission to completion of each emergency procedure was recorded for both groups. Including the total time spent from admission to emergency physician reception (T_0−1_), total time spent from admission to stroke team reception (T_0−2_), total time spent from admission to imaging report out (T_0−3_), total time spent from admission to laboratory report out (T_0−4_), and total time spent from admission to intravenous thrombolysis (T_0−5_).

The clinical outcome indicators were observed for both groups. Including vascular recanalization rate, symptomatic intracerebral hemorrhage incidence, mortality rate.

The National Institutes of Health Stroke Scale (NIHSS) score was evaluated for both groups after the intervention. It was divided into 11 scoring items such as consciousness level and dysarthria. The total score was 42, and the lower the score, the lighter the neurological impairment.

The Barthel score was evaluated for both groups after the intervention. The total score was 100, measuring the patient's self-care in 10 dimensions, such as walking up and down stairs, dressing and bathing. The higher the total score, the less dependent the patient was.

The treatment satisfaction rate of the patients was investigated for both groups. The survey scale was self-administered by the stroke team, with a Cronhach' s α coefficient of 0.84 and a split-half reliability of 0.88, and was surveyed before the patients were discharged. There were four items: health education, basic care, nursing attitude, and nursing skills. The total score of 90~100 was very satisfied, 70~89 was satisfied, 60~69 was generally satisfied, and <60 was dissatisfied. Overall satisfied rate = (very satisfied + satisfied) number of people /total number of people × 100%.

### Statistical Methods

SPSS 22.0 software was used. Enumeration data were expressed as ratios, and the χ^2^ test was used for comparison between groups. Measurement data conforming to normal distribution were expressed as mean ± standard deviation (M ± SD), and *t*-test was used for comparison between groups. Statistical significance was expressed as *P* < 0.05.

## Results

### The Total Time of Each Procedure in the Two Groups of Patients

**Figure 2:**
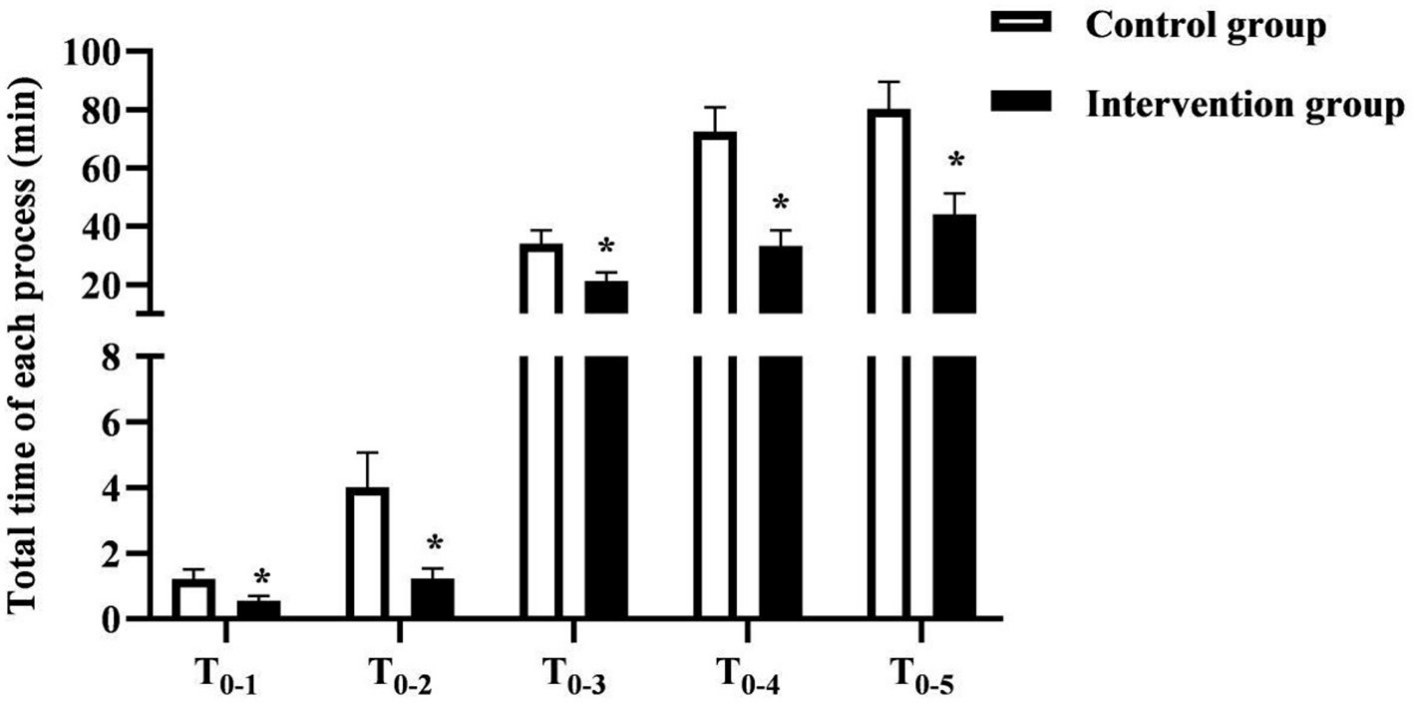
The total time of each procedure in the two groups of patients. * was the comparison of similar items between groups, *P* < 0.05.

The total time of T_0−1_, T_0−2_, T_0−3_, T_0−4_, T_0−5_ in the intervention group (0.55 ± 0.15, 1.23 ± 0.30, 21.24 ± 3.01, 33.30±5.28, 44.19 ± 7.02) min was shorter than that of the control group (1.22 ± 0.28, 4.01 ± 1.06, 34.12 ± 4.44, 72.48 ± 8.27, 80.31 ± 9.22) min (*P* < 0.05). As seen in Figure 2.

### Clinical Outcome Indicators in the Two Groups of Patients

**Figure 3:**
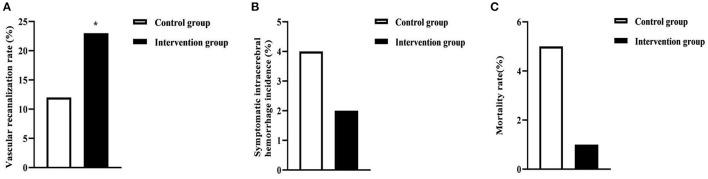
Clinical outcome indicators in the two groups of patients. **(A)** was the vascular recanalization rate. **(B)** was the symptomatic intracerebral hemorrhage incidence. **(C)** was the mortality rate. * was the comparison of similar items between groups, *P* < 0.05.

The vascular recanalization rate in the intervention group (23.00%) was higher than that in the control group (12.00%) (*P* < 0.05). There was no statistical significance in the symptomatic intracerebral hemorrhage incidence and mortality rate in the two groups (*P* > 0.05). As seen in Figure 3.

### NIHSS and Barthel Scores in the Two Groups of Patients

**Figure 4:**
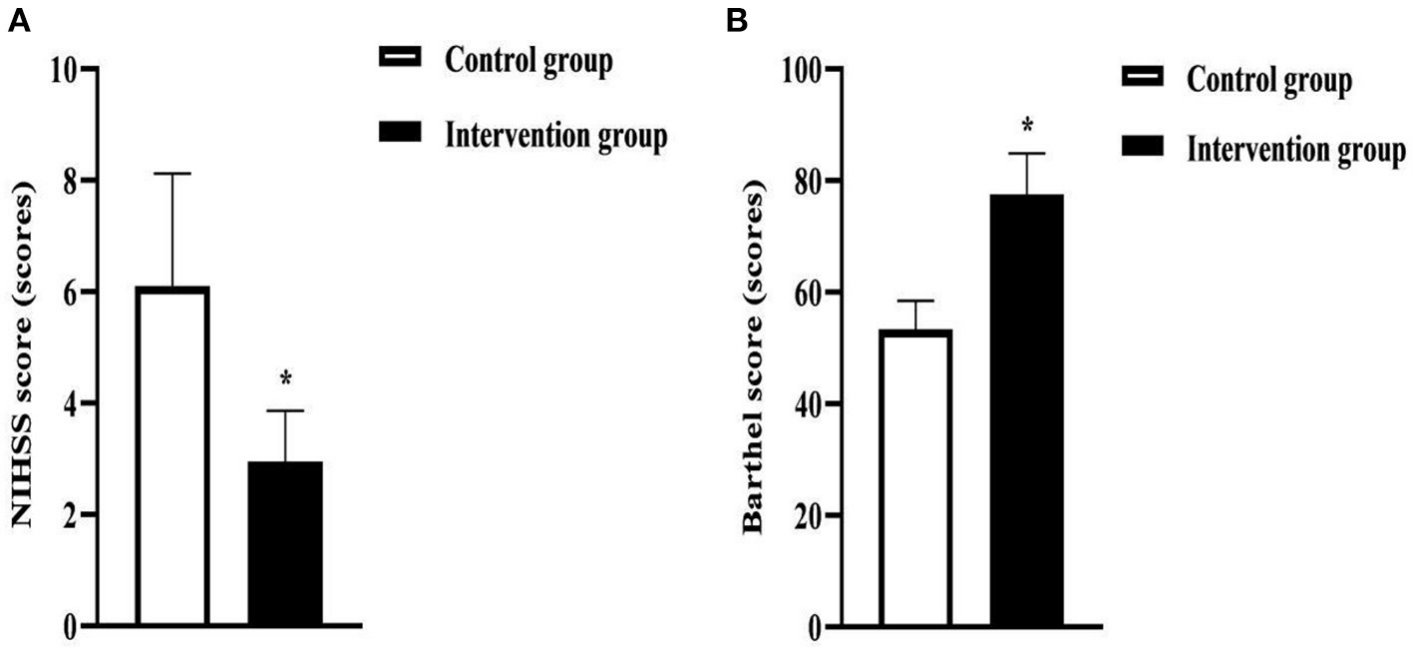
NIHSS and Barthel scores in the two groups of patients. **(A)** was the NIHSS score. **(B)** was the Barthel score. * was the comparison of similar items between groups, *P* < 0.05.

After intervention, the NIHSS score of the intervention group (2.95 ± 0.91) was lower than that of the control group (6.10 ± 2.02), and the Barthel score (77.58 ± 7.33) was higher than that of the control group (53.34 ± 5.12) (*P* < 0.05). As seen in Figure 4.

### The Treatment Satisfaction Rate in the Two Groups of Patients

**Figure 5:**
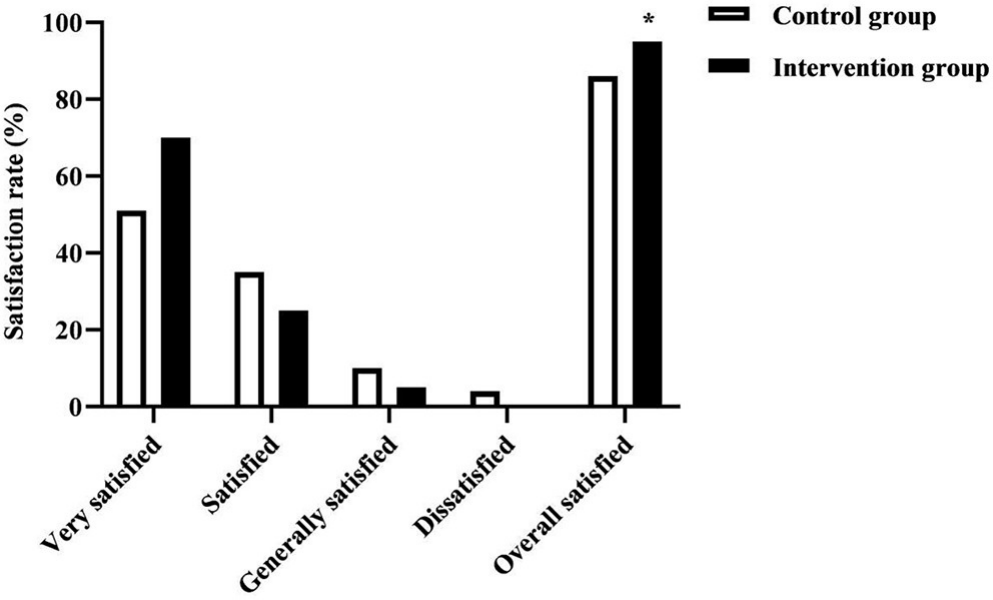
The treatment satisfaction rate in the two groups of patients. * was the comparison of similar items between groups, *P* < 0.05. The intervention group was no dissatisfied.

The treatment satisfaction rate in the intervention group (95.00%) was higher than that of the control group (86.00%) (*P* < 0.05).As seen in Figure 5.

## Discussion

Once AIS patients develop symptoms, the most direct and effective treatment method is early intravenous thrombolysis. For patients with acute onset within 4.5 h, timely and effective thrombolytic therapy can not only effectively reduce the irreversible damage to brain tissue caused by cerebral hemorrhage, but even help patients fully recover to normal levels (15, 16). It is speculated that the degree of prognostic recovery in AIS patients is closely related to the reperfusion time of ischemic brain tissue (17). Based on this, from the onset of the patient to the process of receiving thrombolytic therapy, the waiting time is saved to the maximum extent for the patient, which can create more survival and recovery opportunities for the patient, thereby improving the overall treatment effect. This study focuses on the scientific emergency nursing model, in order to make up for the insufficiency of the current emergency nursing process and improve the clinical treatment efficiency and cure rate of AIS patients by reconstructing the relevant links such as inspection, evaluation, nursing and treatment of AIS patients.

In the results of this study, the total time spent from admission to emergency physician, total time spent from admission to stroke team, total time spent from admission to imaging report, total time spent from admission to test report, and total time spent from admission to intravenous thrombolysis in the intervention group were shorter than that of the control group. It is suggested that the integrated emergency care model based on FMEA can effectively shorten the treatment time of each emergency process of AIS patients. Under the conventional nursing mode, the emergency nursing of AIS patients has problems such as unreasonable treatment process, insufficient understanding of the concept of golden time window by nursing staff, and no green channel. These are the main reasons for the lack of effective treatment or unsatisfactory treatment effect in AIS patients within the golden time window (18, 19). FMEA is a scientific and systematic quality control management method (20). The application of the integrated emergency care model based on FMEA in this study. Through the quantification of RPN, the stroke team identified 6 potential high-risk links in the hospital's emergency procedures. The failure causes and optimization measures are also analyzed. Among them, the training and management of comprehensive quality and competence of nursing staff can help strengthen their knowledge about thrombolysis and improve the shortcomings of previous emergency nurses such as insufficient awareness of time window and improper triage; Advance science and electronic education to patients or their families will help to promote their cooperation and help them make thrombolysis decisions as soon as possible; The establishment of an in-hospital information platform realizes the full time-course sharing of information and data among departments, and solves the problems of message lag, repeated information collection, tedious verification work, and poor communication between doctors, nurses, and patients among departments; The opening of the AIS emergency green channel, expedited appointments for relevant examinations, delivery of examinations and medication pickup by professionally trained nurses, etc. greatly reduce the waiting time and ineffective round trip time for each process; The setting of a special post for thrombolytic nurses reduces the reaction time of patients with thrombolytic drugs; Focused discussion and improvement of problems that arise during the implementation of the optimized process will help ensure continuous improvement in the quality of emergency care.

After the implementation of the integrated emergency care model based on FMEA in this study, it was found through statistical analysis that the vascular recanalization rate of patients increased by 11%. This may be because the construction of an integrated emergency care model based on FMEA has effectively shortened the delay time of each emergency procedure for patients, thus ensuring that as many patients as possible can complete thrombolytic therapy within the treatment time window. In the results of this study, there was no significant difference between the intervention group and the control group in the symptomatic intracerebral hemorrhage incidence and mortality rate. This may be because, even if intravenous thrombolysis is completed within the time window, 6% of patients with AIS are still at risk of symptomatic intracerebral hemorrhage after treatment (21). This further suggests that after the optimization of the emergency procedure in this study, although the overall treatment rate of patients was effectively improved, it was still much lower than that in developed countries such as the United States, and the safety and efficacy of patients receiving thrombolytic therapy still need to be improved.

The results of this study also suggested that the NIHSS score of the intervention group after intervention was lower than that of the control group, and the Barthel score was higher than that of the control group. The treatment satisfaction rate in the intervention group was higher than that in the control group. This suggests that the handover application of the integrated emergency nursing model based on FMEA in AIS patients has certain positive significance and practicability. AIS has acute onset and rapid progression, and early and timely professional emergency care is of great significance to block the progression of the disease, reduce the degree of brain damage, and improve its prognosis (22, 23). In this study, the integrated emergency care model based on FMEA was implemented. The reduction of in-hospital delay time significantly improved the efficiency of emergency care for AIS patients, which allows the majority of patients who are admitted in time to receive thrombolytic therapy within the “time window”, which ensures that the embolized vessels are opened and the blood supply to the ischemic semidark zone tissues is restored within a short period of time after the onset of the disease. The early suppression of disease progression and the significant improvement in vascular recanalization rates resulted in a corresponding improvement in the prognosis of the patients' quality of survival.

## Conclusion

Nursing management is an essential and important link in stroke emergency procedures. Through FMEA, the failure mode that affects the emergency time of AIS patients is effectively analyzed and the targeted optimization process is proposed, which are important to enhance the efficiency and success rate of resuscitation of medical and nursing staff and improve the prognosis and life ability of patients.

## Data Availability Statement

The original contributions presented in the study are included in the article/supplementary material, further inquiries can be directed to the corresponding author/s.

## Ethics Statement

The studies involving human participants were reviewed and approved by the Ethics Committee of The First Affiliated Hospital of Hebei North University. The patients/participants provided their written informed consent to participate in this study.

## Author Contributions

YY and QC are the mainly responsible for the writing of the article. JC is mainly responsible for research design. XZ and QX are mainly responsible for data analysis. AS is responsible for the guidance of the entire research. All authors contributed to the article and approved the submitted version.

## Funding

This study was supported by Zhangjiakou Science and Technology Research Plan Project (No. 1921065D).

## Conflict of Interest

The authors declare that the research was conducted in the absence of any commercial or financial relationships that could be construed as a potential conflict of interest.

## Publisher's Note

All claims expressed in this article are solely those of the authors and do not necessarily represent those of their affiliated organizations, or those of the publisher, the editors and the reviewers. Any product that may be evaluated in this article, or claim that may be made by its manufacturer, is not guaranteed or endorsed by the publisher.
